# The changing scenario of infective endocarditis

**DOI:** 10.1007/s12055-024-01691-z

**Published:** 2024-01-26

**Authors:** Carlos A. Mestres, Eduard Quintana

**Affiliations:** 1 Department of Cardiothoracic Surgery and The Robert WM Frater Cardiovascular Research Institute, The University of the Free State, PO Box 339 (Internal Box G32), Bloemfontein, 9300 South Africa; 2 Department of Cardiovascular Surgery, Hospital Clinic, University of Barcelona, Barcelona, Spain

**Keywords:** Infective endocarditis, Infectious diseases, Diagnosis, Antimicrobial therapy, Surgery

Almost invariably, articles dealing with the complex topic of infective endocarditis (IE) highlight in their introductory remarks that IE is an uncommon disease with a high mortality burden [1]. The medical community thus recognizes that this is a serious and still challenging disease as mortality and frequent morbid events are the norm and not the exception [2]. Looking at IE from any perspective, it seems that rough data with regard mortality, medical and surgical, has not substantially changed since the early reports on surgical treatment, pathophysiology, and chemotherapy [3, 4]. This introductory editorial focuses on discussing the evolution of the status of IE over time.

## Epidemiology

Over the past six decades, we witnessed a number of changes in IE. First, epidemiology has changed. In the last 20 years, the median age of patients has increased and about three-quarters of them have native valve IE and a similar proportion present early in the disease, usually less than 30 days with the classical clinical picture of IE. Recent healthcare exposure was found in one-quarter of patients [5]. In current times, *Staphylococcus aureus* is the most common pathogen. The patient with IE is frequently complicated with stroke and peripheral embolization, heart failure, and intracardiac abscess in varying proportions between 15 and 30%. We operate more patients, with estimates of around one-half of the IE patients undergoing an operation, with an upward trending [6]. However, the toll to pay is that in-hospital mortality for the entire spectrum of IE remains high, in the range of 20–30%. All this has been very elegantly highlighted in the seminal contributions of Murdoch and Ambrosioni and the International Collaboration on Endocarditis-Prospective Cohort Study Group Investigators (ICE-PS) [7, 8], which concluded that IE in the twenty-first century is more an acute disease, with a high rate of *S. aureus* infection and high mortality, which in some regions is trending downwards.

## Diagnostic criteria

The first criteria for the diagnosis of IE of Von Reyn et al., published in 1981, categorized the cases of IE as definite, probable, possible, or the cases were rejected. Other than categorizing, authors used strict definitions that were useful in managing suspect cases [9]. In 1994, the Duke Endocarditis Service proposed an update and the concept of major and minor criteria were introduced. Two major and six minor criteria were established. The diagnostic categories of “definite,” “possible,” and “rejected” were defined, excluding “probable.” The importance of imaging was understood and specific echocardiographic findings were added to the diagnostic armamentarium [10].

Due to changes in microbiology and the consolidation of echocardiography as a fundamental tool in the diagnosis of IE, the modified Duke criteria published in 2000 removed the “possible” echocardiographic category and expanded the inclusion of *Staphylococcus aureus* [11]. Further developments in imaging and nuclear medicine led to the inclusion in the European Society of Cardiology (ESC) 2015 Guidelines for the Management of IE of positron emission tomography (PET)/computed tomography (CT) and other functional imaging modalities as supplementary diagnostic methods in complex or difficult cases [12].

Finally, the International Society of Cardiovascular Infectious Diseases (ISCVID) updated the 2000 diagnostic criteria considering additional changes in microbiology and laboratory techniques and that around 40–50% of the patients will undergo an operation for IE [13]. Therefore, the ISCVID-Duke Criteria now contemplate expanded microbiology, especially the inclusion of *Enterococcus faecalis* and most *Streptococci* among others. Molecular diagnosis is from valve tissue, namely 16S ribosomal RNA (rRNA), polymerase chain reaction (PCR), or fluorescence in situ hybridization (FISH) sequencing and next-generation sequencing from blood. Surgery is now a major criterion. There are currently a number of ongoing studies in different European countries aiming at evaluating their diagnostic accuracy [14].

## Recommendations

According to sources, a “guideline” is information intended to advise people on how something should be done, or what something should be, and a “recommendation” is a suggestion that something is good or suitable for a particular purpose or job and also an advice telling someone what the best thing to do is (https://dictionary.cambridge.org/dictionary/english/). Clinical practice guidelines are an important part of practice worldwide as they give us, the practitioners, an overview of a disease or process, they allow us to produce a more organized approach to them using common pathways by unifying criteria and, in the end, assist in patient care. Fortunately, they are not the law and should never become the law.

There is now a long tradition of guidelines in IE. Over the past three decades, diverse scientific societies have issued specific guidelines and scientific statements covering from the evaluation of anti-infective drugs [15], to prevention of infection by specific pathogens [16], or the management of IE in drug addicts [17]. All these documents produce recommendations with impact at the national level or worldwide. With regard the indication for surgery, congestive heart failure (CHF) due to acute valvular regurgitation, not rapidly controlled or secondary to prosthetic valve dysfunction, is the key indication acknowledged by the Spanish Society of Cardiology in 2000 [18]. The Swedish guidelines for diagnosis and treatment of infective endocarditis of 2007 recommended emergency surgery, on the same day, for acute aortic regurgitation, rupture of a sinus of Valsalva aneurysm into a heart chamber or into the pericardium; and recommended urgent surgery within the first 12 days for valvular obstruction, unstable prosthesis, CHF with patients in New York Heart Association (NYHA) class III–IV, in septal perforation, or evidence of annular abscess or fistula formation [19].

On the other side of the Atlantic, the American Heart Association (AHA) guidelines of 2007 recommended immediate evaluation for surgery in patients with CHF and consideration for surgical intervention when there is echocardiographic evidence of valve dehiscence, perforation, rupture, or fistula or large perivalvular abscess [20]. Once more, the 2015 ESC Guidelines for the Management of IE confirm CHF, uncontrolled infection, and prevention of embolism as the strongest indications for emergency/urgent surgery. The agreement is that CHF due to aortic or mitral native or prosthetic IE with severe aortic regurgitation, obstruction, or fistula causing refractory pulmonary edema or cardiogenic shock as the only likely unavoidable emergency case in IE [21]. All these were also supported by the 2016 American Association for Thoracic Surgery (AATS) consensus guideline in the surgical treatment of IE [22].

The latest guidelines, the 2023 ESC Guidelines for the management of IE, also confirm CHF and uncontrolled infection as a primary indication for surgery [23] and have introduced other substantial modifications in antibiotic prophylaxis, the increasing importance of the endocarditis team, the revision of diagnostic criteria, the disrupting introduction of oral antibiotic treatment, and, when it specifically comes to surgery, the timing for surgical treatment as the latter is, probably for the first time, well defined now [23, 24]. This means that an emergency is an operation undertaken within 24 h of diagnosis, an urgent operation is one that should be performed within 3 to 5 days, and a non-urgent operation is the one done within the same admission [23, 24].

## The rationale behind the special issue on infective endocarditis

All the above show that IE continues to be a medical and surgical challenge. There are still a variety of intriguing aspects of this multifaceted and serious disease. Since the first formal surgical description by Wallace [3], the community has gathered knowledge and experience; we have seen, as described above, an evolution based in the collection and analysis of data, critical thinking, and clinical and experimental work. Older and sicker patients are operated on [6, 25] and the outcomes after a combined and well-structured medical and surgical therapy improved across different subsets of patients [26]. It is even very likely that when it comes to surgery we are close to treatment equipoise [27], as frequently the patients are quite sick and precisely in IE those sicker are proven to have the most benefit from an operation [28]. Other than providing the patient compassionate and prompt care, improved outcomes and quality are major issues as it was elegantly pointed out by the Indian Association of Cardiovascular and Thoracic Surgeons (IACTS) former President Shiv Kumar Nair [29].

This special issue aims at reviewing important aspects of our daily practice, from diagnostic imaging and microbiology, preoperative assessment, intraoperative conduct of a variety of procedures, and postoperative care. A panel of renowned international experts agreed to participate in this editorial undertaking of IACTS by presenting the readership with an updated perspective on this challenging entity.
